# A Pilot Study of Determining the Reliability of a New Three-Dimensional Scanning System for Measuring Truncal Thickness After Breast Cancer Surgery

**DOI:** 10.1089/lrb.2022.0021

**Published:** 2023-04-20

**Authors:** Fumiya Hisano, Sachiyo Watanabe, Shiori Niwa, Keisuke Nakanishi, Ayana Mawaki, Yukari Takeno, Kaoru Murota, Ikumi Honda, Etsuko Fujimoto, Chika Oshima

**Affiliations:** ^1^ Department of Nursing, Nagoya University Graduate School of Medicine, Nagoya, Japan.; ^2^ Japanese Red Cross Nagoya Daini Hospital, Nagoya, Japan.; ^3^ Department of Nursing, Faculty of Nursing, Kansai Medical University, Hirakata, Japan.

**Keywords:** three-dimensional imaging, breast cancer, lymphedema, truncal lymphedema

## Abstract

**Background:**

Lymphedema often affects the trunk after breast cancer surgery. Measuring volume baseline can help detect lymphedema-related changes early, thereby allowing for early intervention efforts. However, there is no quantitative method for detecting truncal lymphedema. As a preliminary investigation into the development of a new method for measuring truncal lymphedema, this study aimed to investigate the reliability and define the minimal detectable change (MDC) in posterior truncal thickness using a three-dimensional (3D) scanning system.

**Methods and Results:**

This observational study included 21 women who had undergone a mastectomy for breast cancer. The 3D images of every subject's trunk were captured by a handheld 3D scanner at two time points. The acquired 3D images were used to calculate the differences in thickness between the affected and unaffected sides at eight points on the trunk. The reliability was determined by checking for agreement between the trials (intraclass correlation coefficient) and by investigating the presence of systematic bias between the measurement error and true value (Bland–Altman analysis). Then, the MDC was calculated. For 14 of the 21 participants, 3D images without missing data at both time points were obtained. Analysis indicated that there was no systematic bias regarding the mean value at the seven body points. Fair-to-excellent reliability was shown at the five points in the middle of the trunk (MDC: 4.14–9.79 mm). The other three points (at the top and bottom of the trunk) had limited reliability.

**Conclusions:**

The 3D scanning system effectively measured the differences in thickness between the affected and unaffected sides of participants' posterior trunks, with fair-to-excellent reliability in the middle of the trunk.

## Introduction

After breast cancer treatment, 8%–56% of patients suffer from breast cancer-related lymphedema.^1^ While upper limb lymphedema is a well-documented problem after breast cancer treatment, there is less information about the occurrence and severity of breast cancer-related truncal edema (i.e., in the chest, shoulder, and back).^2^ Few studies on this topic suggest that truncal lymphedema is associated with painful and nonpainful edema, shoulder discomfort, feelings of fullness and heaviness, and low back pain.^3,4^ Given these symptoms, truncal lymphedema can lessen a patient's ability to function and the quality of life.

Early detection of the problem is important for treating lymphedema effectively, as recent studies have shown that early intervention may prevent the condition's progression to an irreversible stage.^5,6^ Taking volume baseline measurements can help detect lymphedema-related changes early, thereby allowing for early intervention efforts.

While there are several methods for measuring upper limbs (e.g., water displacement and circumference measurement), there is no established quantitative method of detecting truncal lymphedema volume. Furthermore, methods that rely on circumferential measures to assess lymphedema are not appropriate for truncal lymphedema.^7^ Therefore, there is a strong need to develop an accurate measurement method for truncal edema.

Recently, three-dimensional (3D) scanning systems have emerged as a promising tool for measuring limb volume.^8–11^ These noninvasive devices, which enable the real-time digital reconstruction of 3D objects, have shown a high degree of accuracy.^12^ Furthermore, the software's ability to use acquired 3D images to calculate the length and thickness of a particular body part may make it possible to detect size differences between the affected and unaffected sides of the trunk of the body.

As a preliminary investigation into the development of a new method for measuring truncal lymphedema, the purpose of this study was to investigate the reliability and to define the minimal detectable change (MDC) in the thickness of the posterior trunk (dorsal to the midaxillary line) using a 3D scanning system.

## Materials and Methods

### Research design

This pilot cross-sectional observational study was conducted from February 2019 to June 2021.

### Participants

Twenty-one women who had undergone a unilateral mastectomy for breast cancer participated in this study. These women were recruited from among those who were referred to the surgery outpatient units of either the Nagoya University Hospital or the Japanese Red Cross Nagoya Daini Hospital. All the participants were assessed for eligibility according to two criteria. First, they had received a primary and adjuvant breast cancer treatment (e.g., chemotherapy or radiation therapy) that was completed at least 6 months before the study. Those receiving ongoing hormonal therapy were allowed to participate. Second, the women's physician had to permit study participation.

Characteristics of participants (age, weight, and years since surgery) were collected from their medical records. Affected and unaffected arm circumferences were measured by tape measurement at the wrist, 5 cm below the elbow (forelimb), and 10 cm above the elbow (upper limb).

### Ethical considerations

This study was conducted in accordance with the Declaration of Helsinki as revised in 2013. The Ethics Committees of Nagoya University (18-138, 2018-0280-2) and the Japanese Red Cross Nagoya Daini Hospital (1323) approved this study. Patients who participated in the study were explained the purpose of the study and signed a written informed consent.

### Capturing 3D images

Posterior truncal thickness was measured using a handheld 3D scanner (Go!SCAN50™; Creaform). This handheld 3D scanner is based on structured light and the scanner emits a white light pattern. The surface is captured while moving the handheld scanner over the object. A lamp guidance system helps to find the correct scanning distance. Each of the three cameras observes the distortion of the scanned object's pattern and records the calculated color information. According to the manufacturer, the scanner works with a rate of 550,000 measurements per second and a scanning area of 380 × 380 mm with a resolution of 0.5 mm and point accuracy of up to 0.1 mm.^13^

Before scanning, marks were made on the sternal ends of participants' clavicles using a nonpermanent skin marker pen. During scanning, participants stood on the positioner used for scoliosis mass-screening tests (Positioner, A-and-A corporation) while gripping onto the belt (which set the elbow joint with the arm bent 90°) to keep their body immobile. The operator manipulated the 3D scanner to illuminate the entire trunk circumference to capture the desired image data. The image was captured two times, with a 5-minute interval between captures, during which participants stepped off the positioner. Three operators (A, B, and C) participated in image capturing at random. Each operator practiced the image capturing process five times before working with study participants.

### Calculating thickness from 3D images

**FIG. 1.**
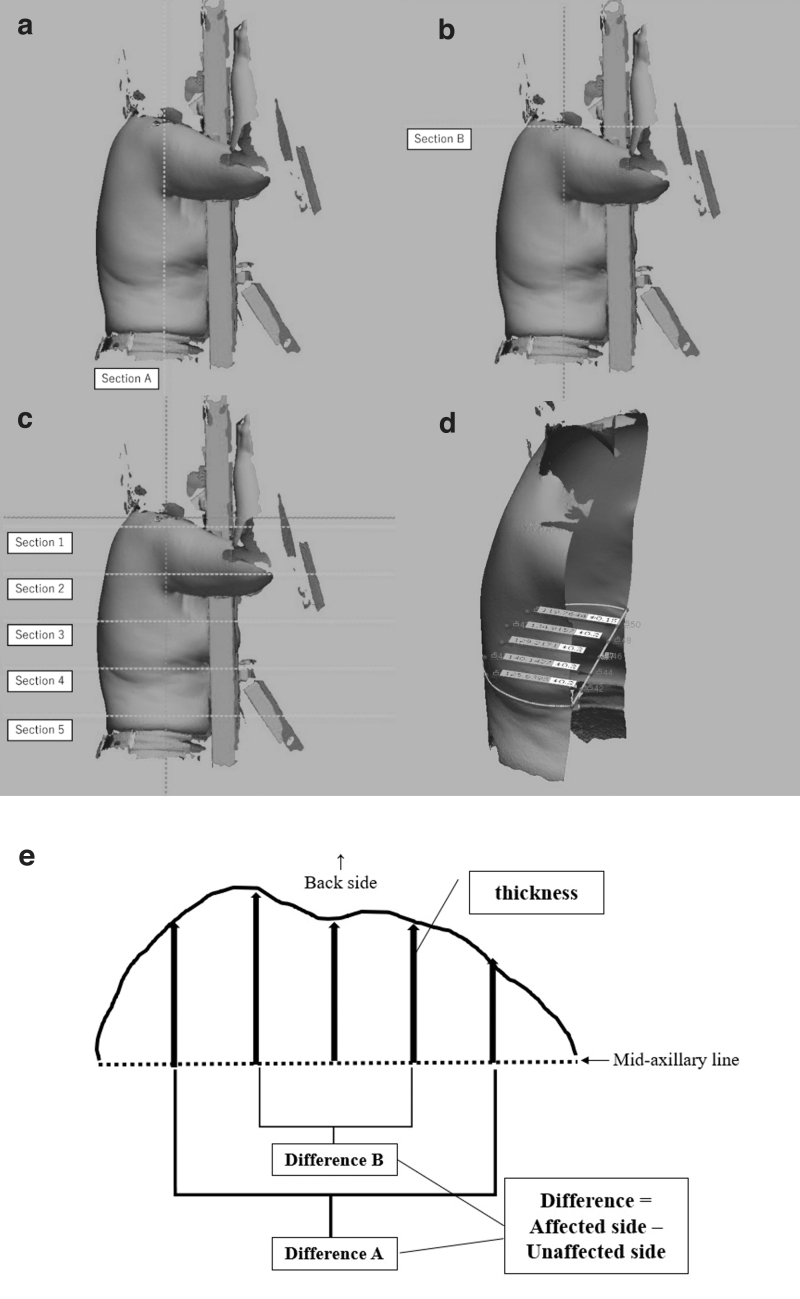
The methods of calculating thickness from 3D images. **(a)** (Step one): Each rater set section A at the coronal plane over the left and right midaxillary lines. All the following steps were performed semiautomatically by the software. **(b)** (Step two): Section B was set perpendicular to section A at shoulder height. **(c)** (Step three): Sections 1–5 were set parallel to section B and set at four equidistant points between the midpoint of the left and right clavicle sternal ends to the midpoint between the left and right anterior superior iliac spine. Only sections 1–4 were used for analysis. **(d)** (Step four): The thickness of the back was measured at five points extending in the dorsal direction originating from the line connecting both axillary medians, which was divided into six equal parts in each cross section. **(e)** (Step five): The difference between the thicknesses of the outer (the two lateral most lines: Difference A) and the inner (the two more medial lines: Difference B) was calculated for each cross section. 3D, three dimensional.

Two raters (A and D) computed the thickness measurements for each 3D image. A dedicated 3D inspection and metrology software (Geomagic^®^ Control X™; 3D Systems) calculated the thickness values through the five-step process described below (Fig. 1).

Step one: Each rater set section A at the coronal plane over the left and right midaxillary lines (Fig. 1a).

All the following steps were performed semiautomatically by the software.

Step two: Section B was set perpendicular to section A at shoulder height (Fig. 1b).

Step three: Sections 1–5 were set parallel to section B and set at four equidistant points between the midpoint of left and right clavicle sternal ends to the midpoint between the left and right anterior superior iliac spine. Only sections 1–4 were used for analysis (Fig. 1c).

Step four: The thickness of the back was measured at five points extending in the dorsal direction originating from the line connecting both axillary medians, which were divided into six equal parts in each cross section (Fig. 1d).

Step five: The difference between the thicknesses of the outer (the two lateral most lines: Difference A) and the inner (the two more medial lines: Difference B) was calculated for each cross section. The difference was calculated by subtracting the unaffected side value from the affected side value (Fig. 1e). In other words, the differences were calculated at eight points: two points (Differences A and B) for each section 1–4.

### Statistical analysis

Before determining this measuring method's reliability, it was necessary to clarify the agreement of the results from calculating thickness measurements from 3D images between raters. The intraclass correlation coefficient (ICC) was calculated to assess this agreement. ICC (1, 1) and ICC (1, 3) were used to determine intrarater reliability, and ICC (2, 1) was used to determine inter-rater reliability. According to Fleiss criteria, an ICC >0.75 indicates excellent reliability, and values between 0.4 and 0.75 represent fair-to-good reliability.^14^

Next, the reliability of the two measurements was determined. The ICC was calculated to assess this agreement of two measurements. Bland–Altman analysis was used to calculate systematic bias, including fixed and proportional biases, in the means for the first and second measurements.^15^ When zero falls within the 95% confidence interval, there is no significant fixed bias. When no correlation is shown between the two measurements, there is no significant proportional bias. Measurement errors and minimal detectable change at the 95% confidence level (MDC95) were calculated only in the absence of systematic bias because this affects the validity of the measurement.

Measurement errors were evaluated using the standard error of measurement (SEM).^16^ The SEM enabled us to interpret the magnitude of the within-subjects variation, which was calculated using Equation (1), in which *Sp* is the pooled standard deviation (SD) of two measurements:


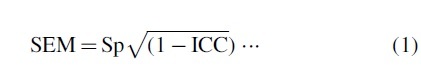


The absolute MDC95 also was calculated to determine the magnitude of change that must occur before the change exceeded the measurement error and variability at the 95% confidence level. Equation (2) was used for this calculation^17^:

(2) $$MDC95=1.96\times SEM\times \sqrt{2}\cdot \cdot \cdot$$

All calculations were performed using SPSS version 27 (IBM, SPSS Statistics).

## Results

### Sample characteristics

Table 1 shows the sample characteristics. Twenty-one patients participated in this study. Twelve of the participants were affected on their right sides. The mean participant age (±SD) was 59.1 ± 10.5 years. The mean participant body weight (±SD) was 53.2 ± 9.9 kg. The mean years (±SD) since surgery was 3.4 ± 3.0 years. The mean (±SD) difference between arm circumferences was 4.6 ± 9.7 mm in the wrist, 5.9 ± 10.0 mm in the forearm, and 4.9 ± 10.2 mm in the upper arm.

**Table 1.** Sample Characteristics

Affected side (right/left,* n*)	12/9		
	Mean* ± *SD	Minimum	Maximum
Age (years)	59.1 ± 10.5	39	85
Weight (kg)	53.2 ± 9.9	39.9	78.1
Body mass index (kg/m^2^)	22.1 ± 3.5	16.7	30.1
Years since surgery	3.4 ± 3.0	0	11
Difference between arm circumferences (mm)			
Wrist	4.6 ± 9.7	−21.0	25.4
Forelimb	5.9 ± 10.0	−12.7	26.7
Upper limb	4.9 ± 10.2	−16.2	28.5

SD, standard deviation.

### Intrarater and inter-rater agreement of calculated thickness

Tables 2 and 3 present the data from the intrarater and inter-rater assessments. Excellent intrarater reliability was observed (all intrarater ICCs >0.88, *p* < 0.01), and inter-rater reliability was fair to excellent (all inter-rater ICCs >0.70, *p* < 0.01).

**Table 2.** Intrarater Assessments

	Mean* ± *SD (mm)		ICC (1, 1)	95% Coefficient interval	*p*
	Trial 1	Average			
Section 1					
Difference A	−0.17 ± 5.89	−0.32 ± 5.33	0.946	0.893 to 0.976	<0.01
Difference B	−1.05 ± 4.30	−0.80 ± 4.23	0.885	0.781 to 0.947	<0.01
Section 2					
Difference A	3.42 ± 7.30	3.30 ± 6.75	0.899	0.806 to 0.954	<0.01
Difference B	0.88 ± 3.86	0.89 ± 3.70	0.918	0.841 to 0.963	<0.01
Section 3					
Difference A	3.27 ± 6.79	3.72 ± 6.17	0.929	0.861 to 0.968	<0.01
Difference B	2.29 ± 3.96	2.48 ± 3.82	0.953	0.907 to 0.979	<0.01
Section 4					
Difference A	1.09 ± 5.47	1.04 ± 5.30	0.963	0.926 to 0.983	<0.01
Difference B	0.68 ± 4.06	0.85 ± 4.05	0.929	0.866 to 0.967	<0.01

ICC, intraclass correlation coefficient.

**Table 3.** Inter-Rater Assessments

	Mean ± SD (mm)		ICC (2, 1)	95% Coefficient interval	*p*
	Rater 1	Rater 2			
Section 1					
Difference A	−0.17 ± 5.89	0.26 ± 4.87	0.818	0.605 to 0.922	<0.01
Difference B	−1.05 ± 4.30	−0.88 ± 4.89	0.708	0.403 to 0.871	<0.01
Section 2					
Difference A	3.42 ± 7.30	4.02 ± 8.70	0.732	0.447 to 0.882	<0.01
Difference B	0.88 ± 3.86	0.83 ± 3.72	0.825	0.615 to 0.925	<0.01
Section 3					
Difference A	3.27 ± 6.79	4.37 ± 6.27	0.848	0.667 to 0.935	<0.01
Difference B	2.29 ± 3.96	2.69 ± 3.83	0.882	0.736 to 0.950	<0.01
Section 4					
Difference A	1.09 ± 5.47	1.27 ± 4.67	0.860	0.686 to 0.941	<0.01
Difference B	0.68 ± 4.06	0.96 ± 4.37	0.887	0.748 to 0.952	<0.01

### Systematic bias in the mean

**FIG. 2.**
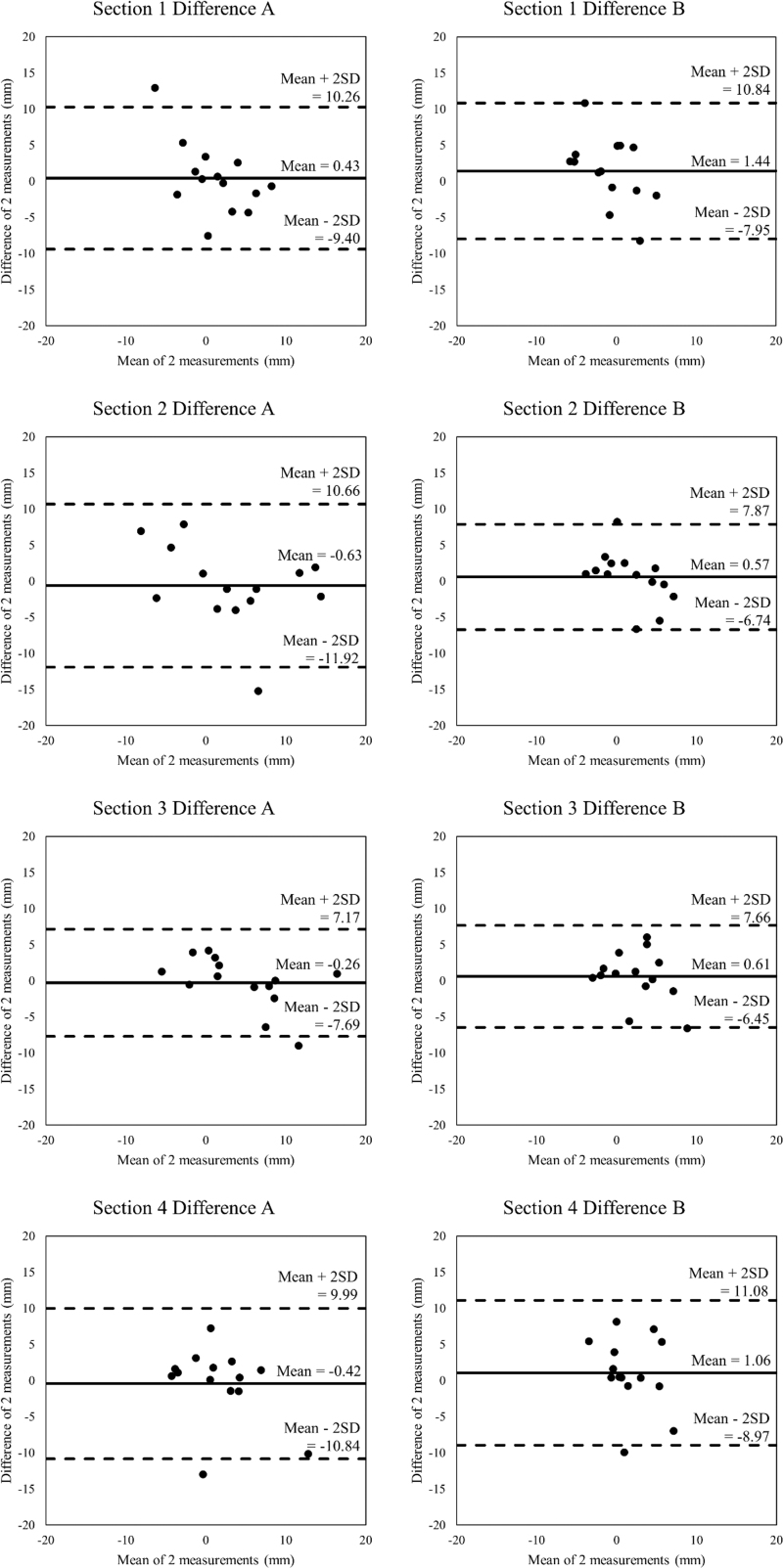
Bland–Altman plots of two trials in all eight points.

Some obtained 3D images exhibited a partial data loss due to the light not reaching certain areas or being over-reflected from other areas. Therefore, we obtained 3D images without missing data at both the time points for 14 of the 21 participants. Bland–Altman analyses indicated that the zero fell within the 95% confidence interval; therefore, there was no significant fixed bias in all eight points for the two measurements (Fig. 2). A correlation was shown only in Difference A in section 1; therefore, there was significant proportional bias. There were no correlations at the other seven points.

### Measurement errors and MDC

Table 4 presents the data for two measurements in each 8-point ICC, systematic bias, 7-point SEM, and MDC95. SEM and MDC95 were calculated only for the seven points that showed no systematic bias.

**Table 4.** Intraclass Correlation Coefficient, Measurement Errors, and Minimal Detectable Change of Two Measurements

	Mean* ± *SD (mm)	ICC	95% Coefficient interval	*p*	Systematic bias	SEM (mm)	MDC95 (mm)
Section 1, *n* = 14							
Difference A							
Test	0.94 ± 5.79	0.487	−0.019 to 0.799	0.03	Proportional bias	—	—
Retest	1.37 ± 3.36						
Difference B							
Test	−1.61 ± 4.95	0.334	−0.201 to 0.721	0.12	No bias	3.83	10.62
Retest	−0.18 ± 3.02						
Section 2, *n* = 14							
Difference A							
Test	3.47 ± 8.57	0.743	0.384 to 0.909	<0.01	No bias	2.86	7.93
Retest	2.85 ± 6.62						
Difference B							
Test	1.44 ± 4.62	0.582	0.113 to 0.842	0.01	No bias	2.36	6.54
Retest	2.01 ± 3.04						
Section 3, *n* = 14							
Difference A							
Test	4.56 ± 7.21	0.838	0.581 to 0.945	<0.01	No bias	1.49	4.14
Retest	4.31 ± 5.31						
Difference B							
Test	2.16 ± 4.34	0.607	0.151 to 0.853	0.01	No bias	2.21	6.13
Retest	2.77 ± 3.42						
Section 4, *n* = 14							
Difference A							
Test	1.87 ± 6.08	0.540	0.053 to 0.823	0.02	No bias	3.53	9.79
Retest	1.45 ± 4.36						
Difference B							
Test	1.21 ± 4.33	0.188	−0.346 to 0.637	0.24	No bias	4.52	12.51
Retest	2.27 ± 3.42						

MDC95, minimal detectable change at the 95% confidence level; SEM, standard error of measurement.

For the Difference A of sections 2, 3, and 4, the agreement of two measurements was fair to excellent (ICCs >0.540, *p* < 0.02). The ICCs for Difference A in sections 2 and 3 were especially high. SEMs in this area ranged from 1.49 to 3.53 mm. The MDC95 data for the Differences A and B of sections 2 and 3, and the Difference A of section 4 ranged from 4.14 to 9.79 mm. The agreement of two measurements for the Difference B of sections 1 and 4 was poor. The MDC95 data were 10.62 and 12.51 mm, respectively, in this area.

## Discussion

Although truncal lymphedema can cause physical and psychological sequelae, there is no quantitative method to detect it. This study was the first to use a handheld 3D scanning system to detect truncal edema in a sample of breast cancer survivors.

First, we clarified the agreement of results between raters. In calculating posterior truncal thickness from the 3D images, excellent intrarater reliability and fair-to-excellent inter-rater reliability were observed. These results showed that one person could get an accurate value in one calculation.

We also found that there was no systematic bias in the mean value for Difference B in section 1 and the mean for Differences A and B of sections 2, 3, and 4, except for the Difference of section 1. In sections 1 and 4, it may be difficult to recreate the same fixed posture because no supporting bone (e.g., the costae) contacted the positioner. This finding indicates that the detection in these areas (sections 1 and 4) requires improvements. However, because truncal lymphedema frequently occurs around the axillary region (i.e., chest, shoulder, and back) that corresponds to sections 2 and 3, it is important to accurately measure these two sections to achieve effective lymphedema management.

Our results showed the reliability of measurement in sections 2 and 3 to be fair to excellent. In this area, no systematic bias was shown and MDC95 data ranged from 4.14 to 7.93 mm. The MDC95 represents the smallest change in a score, which is likely to reflect the true change, rather than the measurement error alone.^17^ This finding suggests that these measurements can provide basic data for developing novel methods for measuring truncal lymphedema.

This study focused on the posterior truncal thickness. However, truncal edema may occur in the anterior trunk, such as the breast or anterior thorax. Therefore, further studies are required to develop accurate measurement methods of the anterior trunk area.

### Limitations

Although our results demonstrated high reliability in sections 2 and 3, our study targeted patients before lymphedema onset and those with a short onset period. Therefore, patients with long-term and significant edema may have different results. Further studies with more patients are required to confirm these results.

## Conclusions

The 3D scanning system measured the differences in thickness between the affected and unaffected sides of participants' posterior trunks, with fair-to-excellent reliability in the middle of the trunk.
